# Genetic variability and intra-genotype recombination of DuCV from ducks and geese in central and north China

**DOI:** 10.3389/fvets.2026.1775879

**Published:** 2026-04-13

**Authors:** Haoyang Wang, Yuzhu Dong, Xiaomin Hu, Jinglan Wang, Xin Xu, Dandan Li, Jun Ji, Lunguang Yao, Yingzuo Bi, Qingmei Xie

**Affiliations:** 1 Henan Provincial Engineering Laboratory of Insects Bio-reactor, Henan Provincial Engineering, and Technology Center of Health Products for Livestock and Poultry, Henan Provincial Engineering and Technology Center of Animal Disease Diagnosis and Integrated Control, Nanyang Normal University, Nanyang, China; 2 Langfang Academy of Agricultural and Forestry Sciences, Langfang, China; 3 College of Animal Science, South China Agricultural University, Guangzhou, China

**Keywords:** duck circovirus, ducks, geese, phylogenetic analysis, recombination analysis

## 1 Introduction

According to the latest report from the International Committee on Taxonomy of Viruses (ICTV), the *Circoviridae* family comprises two genera of *Cyclovirus* and *Circovirus* (1). Duck circovirus (DuCV), which belongs to the genus circovirus, is the non-enveloped, icosahedral virus with a diameter of 15 to 16 nm (2–4). The DuCV's genome is a ~2 kb single-stranded circular DNA harboring three open reading frames (ORFs): ORF1 encodes the virus replicase, ORF2 encodes the major antigenic capsid protein, and the ORF3 encodes apoptosis-related protein (5–7). Furthermore, the genomic region between the ORF1 and ORF3 harbors a stem-loop that acts as a cis-acting element for viral replication initiation (8). To date, three main genotypes of the DuCV have been classified: DuCV-1 (1a–d), DuCV-2 (2a–c), and DuCV-3 which was first discovered in China in 2022 (9). The DuCV-1/2 may evolve from the genetically similar ancestors through different pathways. Despite being the most recently identified, DuCV-3 exhibits a relatively low genomic identity (62.3%−63.7%) with other known DuCVs (9).

DuCV was initially found in two 6-week-old female ducks from a German farm in 2003 and has since spread worldwide (8). In China, the virus was first reported in Taiwan province in 2006, and its infection has since been detected in all poultry-raising regions (10). According to accumulating evidence, DuCV could infect the ducks of all breeds and ages, mainly affect the immune system negatively and cause severe immunosuppression, thereby making infected ducks more susceptible to secondary infections (11–14). Clinically, infected ducks exhibit irregular plumage, stunted growth, lymphocytopenia, and hepatic/splenic necrosis (15). The pathogenic characteristics of the DuCV are systemic infection, persistent infection, and horizontal transmission (16, 17). Emerging investigations have documented the detection of DuCV in Chinese geese, indicating an expansion of its host range (15). Therefore, persistent epidemiological surveillance of DuCV in duck and goose populations is of significant clinical importance for understanding and controlling DuCV infection.

## 2 Material and methods

### 2.1 Virus investigation

From January 2024 to July 2025, 750 tissue samples were collected from birds exhibiting the clinical signs of extensive dorsal feather loss and lethargy from 75 farms, including 23 farms (8 duck farms and 15 goose farms) from Hebei Province, 23 farms (6 duck farms and 17 goose farms) from Hubei Province, and 29 farms (10 duck farms and 19 goose farms) from Henan Province. All the birds used for sampling died from cachexia or infection of unknown cause. All the farms were single species farms, exclusively raised either geese or ducks, and were not in contact with other farms. Due to the absence of specific clinical signs in some waterfowl infected with DuCV, a sampling strategy was employed wherein 10 birds were selected from each farm. All birds were transported to the laboratory under refrigerated conditions for necropsy. Stringent measures were implemented during sample processing to prevent cross-contamination between individual birds.

### 2.2 Sample processing and Viral DNA extraction

Liver and spleen tissues from each bird were collected, and equal masses (500 mg each) of the two tissue types were combined and then homogenized. After snap-freezing in liquid nitrogen and grinding, 20 mg of the resulting powder was taken, and viral nucleic acid was extracted using the Virus DNA/RNA Isolation Kit (YALEPIC BIOTECH, Suzhou, China) according to the manufacturer's instructions. After quantifying with the NanoDrop ND-1000 spectrophotometer (Thermo Fisher Scientific, Waltham, MA, United States), the extracted viral DNA was stored at −80 °C until further use.

### 2.3 Amplifying by PCR and sequencing

PCR was performed according to established methods as described in previous study (15). All samples were screened for DuCV by PCR using extracted DNA as the template. Two primer sets were employed: a universal primer set (DuCV12-F1:5′-AATACACAGACCCACCGGCC3′ and DuCV12-R1:5′-CGTACCTTCACCCGCTCCTT3′) for both DuCV-1 and DuCV-2 (16); and a specific primer set (DuCV3-F1:5′-GCTGATCGTCGAAACGCAACGTG3′ and DuCV3-R1:5′-TGTCGACTCCTTTGCACAATATTC3′) specific for DuCV-3 (9). Furthermore, full-length genome amplification was performed on positive DNA samples using a back-to-back primer pair (DuCV-F2: 5′-CTSTCTCGWGCYCGGGGATCTGAC-3′ and DuCV-R2: 5′-CCAGGCTCTTCCTCCCAGCKWCTCTT-3′), previously reported to amplify the full-length genomes of both DuCV and GoCV as universal primers (18). All cycling conditions for each PCR assay were listed in Supplementary Table 1. Subsequently, the PCR amplicon with correct molecular weight (complete genome) as visualized by gel electrophoresis was cloned into the pMD-18T vector and submitted for Sanger sequencing (Tongyong, Anhui, China). The obtained whole genome sequences were assembled via the SeqMan modules (DNASTAR, WI, US) using the default settings and verified based on the sequence-similarity through BLAST (Basic Local Alignment Search Tool) in NCBI database.

### 2.4 Phylogenetic and recombination analysis

To understand the evolutionary relationship between these newly identified DuCVs and other known strains, genome sequences of the new DuCVs and 104 reference sequences retrieved from the NCBI database were subjected to multiple sequence alignment using the ClustalW method via Molecular Evolutionary Genetics Analysis (MEGA) v.10.2.4 (19). Phylogenetic trees were constructed using the maximum likelihood (ML) method based on the TN93+G substitution model, with branch support evaluated by 1,000 bootstrap replicates.

Given DuCV's well-documented propensity for recombination, the genome sequences of the newly obtained and 104 reference strains representing different genotypes were analyzed using RDP version 4.83 with seven distinct detection methods (RDP, GENECONV, Bootscan, Maximum Chi-Square, Chimera, SISCAN, and Distance Plot). Putative recombination events identified by RDP (v4.83) were further analyzed using SimPlot v3.5.1 software to validate recombination breakpoints and characterize recombination patterns using bootscan analysis (15).

## 3 Descriptive results

### 3.1 Sample screening

Through DuCV screening tests, 9 out of 24 duck farms (the positivity rate for birds within each farm ranged from 40% to 100%), and 4 out of 51 goose farms tested positive for DuCV (the positivity rate for birds within each farm ranged from 20% to 40%). The provincial distribution showed 5 positive farms in Henan (4 duck farms and 1 goose farm), 4 in Hubei (2 duck farms and 2 goose farms), and 4 in Hebei (3 duck farms and 1 goose farm). These results suggested that DuCV can infect not only ducks but also geese, which was supported by both this study and our prior work (15). By suppressing the host immune system, DuCV predisposes infected birds to secondary infections and increases their susceptibility to other viral pathogens (20, 21). This exacerbates clinical disease severity, leading to significant economic losses in the poultry industry and implementing preventive measures against DuCV is recommended (11). In addition to our findings regarding the expanding host diversity of DuCV, infection in wild ducks has also been documented in other studies (22). Therefore, epidemiological investigation in wild waterfowl and other poultry is warranted to prevent the complex and extensive transmission of DuCV (2). Given that DuCV is known to be transmitted horizontally among birds, rigorous monitoring of water and feed sources is essential to prevent exposure to contaminated materials (17). Concurrently, implementing regular health surveillance and prompt isolation of sick or deceased birds are critical measures to contain viral spread within flocks.

### 3.2 Phylogenetic and gentyping analysis

**Figure 1:**
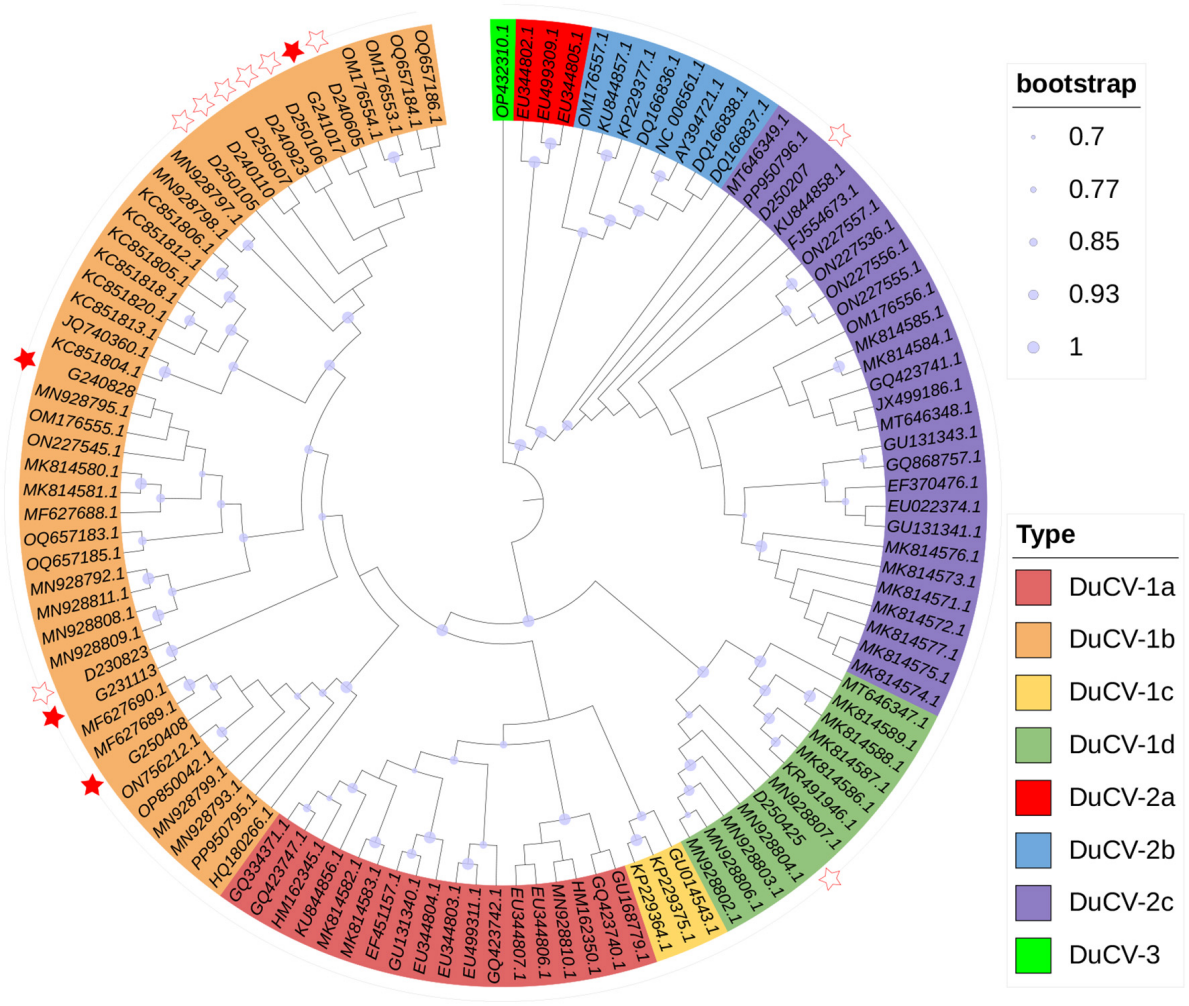
Phylogenetic tree constructed from complete genomes of 13 identified DuCV strains and reference sequences. Solid circles on the branches represented bootstrap values. Colored ranges represented different genotype classifications. The sequences identified in this study were marked with stars: goose-derived strains were indicated by a solid red pentagram, and duck-derived strains were indicated by an open pentagram.

Referring to sequencing results, all the DuCV-positive samples from the initial screening were also positive for the full genome screening. The nucleotide sequences obtained from all positive samples within the same farm exhibited 100% similarity. The phylogenetic tree based on whole-genome alignments of the 13 newly-obtained sequences (Supplementary Table 2) and 104 reference sequences (Supplementary Table 3) is displayed in Figure 1. All the strains were classified into three main DuCV clades (DuCV-1, DuCV-2, and DuCV-3), each further divided into multiple subclades representing different subgenotypes. Phylogenetic analysis revealed that 11 of the 13 sequenced strains belonged to DuCV-1b. The remaining two duck-derived DuCV strains were identified as DuCV-1d (from Hebei Province) and DuCV-2c (from Hubei Province). Notably, all DuCV strains detected in geese belonged to the DuCV-1b genotype. Interestingly, in the phylogenetic tree, goose-derived DuCV strains did not form a distinct clade but were scattered among various DuCV-1b subclades. This pattern provides evidence for the close genetic relatedness between goose-derived and duck-derived DuCV variants. Previous reports indicated that the earliest documented goose-derived DuCV strains belonged to genotypes of DuCV-1b and DuCV-2c (15). In line with this, this study confirmed the presence of DuCV-1b in geese again. Furthermore, concurrent research has established that the predominant genotypes circulating in ducks are DuCV-1b and DuCV-2c (2). Collectively, these reports reasonably supported the inference that duck-derived and goose-derived DuCV shared a high degree of sequence similarity (2, 15).

### 3.3 Recombination analysis

**Figure 2:**
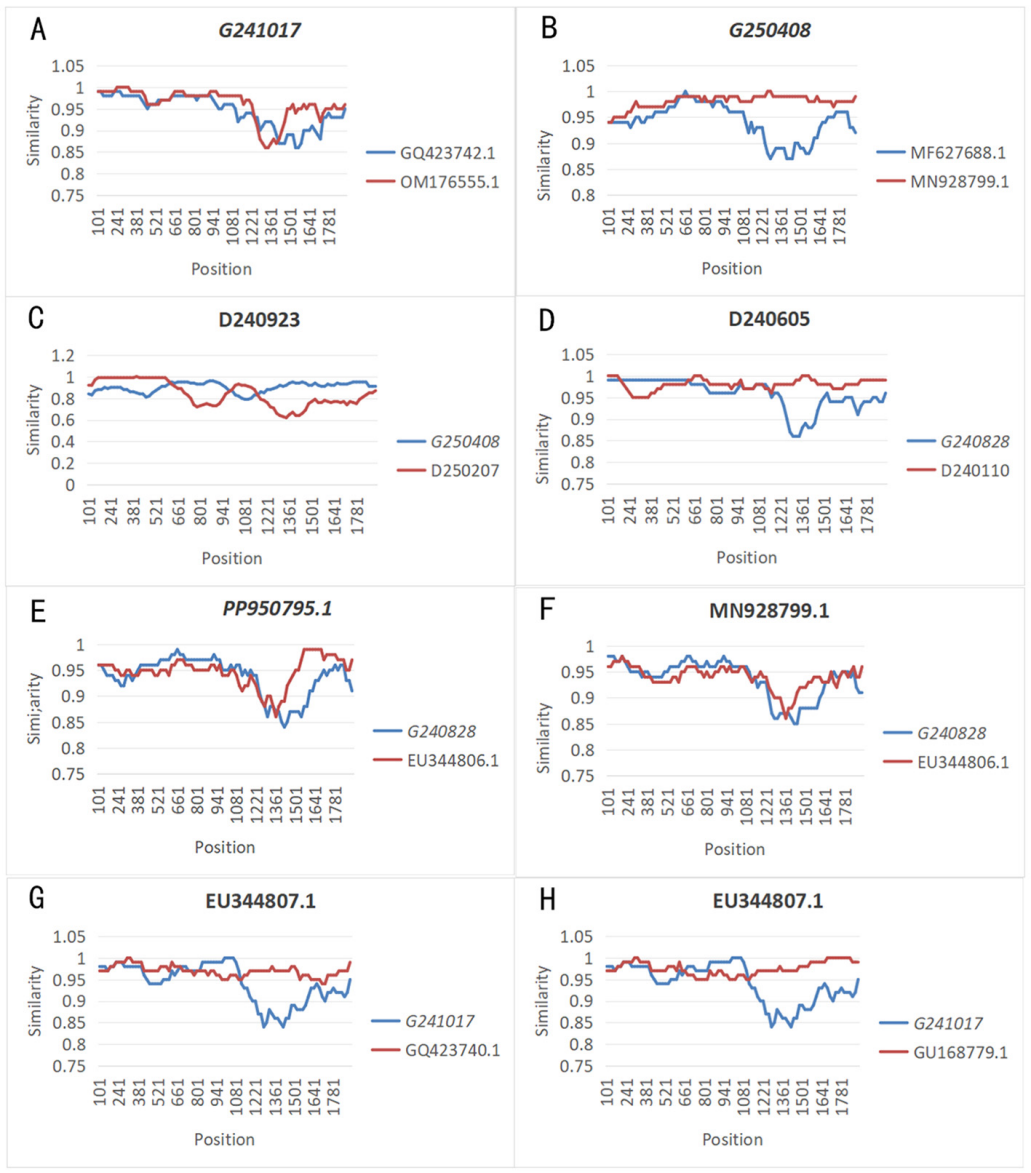
Recombination events were analyzed using SimPlot software for strains G241017, G250408, D240605, PP950795.1, MN928804.1, MN928799.1, and EU344807.1. Italicized text denoted goose-derived DuCV strains. The analysis identified recombination events in: **(A)** G241017, **(B)** G250408, **(C)** D240605, **(D)** PP950795.1, **(E)** EU344807.1, **(F)** MN928804.1, **(G)** MN928799.1, and **(H)** EU344807.1.

Recombination is regarded as the principal driving force behind viral evolution and the primary origin of the majority of viral genetic diversity (23). Based on recombination analysis according to the complete genome sequences of the newly obtained and reference DuCV strains, a total of 17 recombination events were predicted. Notably, among them, 10 were related to DuCV strains identified in geese. Figure 2 presented that the majority of these goose-origin strains participated as major/minor parents participated in recombination events with duck-derived strains. In contrast, the remaining two goose-origin strains (G240117/DuCV-1b and G250408/DuCV-1b) were recombinants from duck-derived DuCV strains (GQ423747.1/DuCV-1a, OM176555.1/DuCV-1b, MF627688.1/DuCV-1b, and MN928799.1/DuCV-1b) identified as their parental donors. These intra-genotype and cross-species recombination events collectively contribute to the genetic variability of DuCV. Owing to the lack of a cell culture system for propagating the requisite virus amounts *in vitro*, the isolation of strains from different host species and the execution of challenge experiments are both restricted (15). Therefore, phylogenetic analyses and predicted recombination events can only serve as preliminary references for inferring cross-host transmission.

In conclusion, this study demonstrates a relative high genetic identity between goose- and duck-derived DuCV strains. The recombination events observed between duck- and goose-derived strains of different genotypes also further hint at the potential horizontal transmission between duck and goose populations. However, the underlying transmission characteristics still require confirmation through extensive epidemiological investigations and experimental infection studies.

## Data Availability

The datasets presented in this study can be found in online repositories. The names of the repository/repositories and accession number(s) can be found below: https://www.ncbi.nlm.nih.gov/genbank/, PX308131 to PX308133.

## References

[1] Dong HV Trinh DQ Tran G Vu TT Nguyen T Rattanasrisomporn A . Characterization of an emerging recombinant duck circovirus in Northern Vietnam, 2023-2024. Viruses. (2025) 17:732. doi: 10.3390/v17050732 40431743 PMC12115742

[2] Li P Zhang F Bao C Liu H Yu K Zhu H . Epidemiological investigation and analysis of the genetic evolution of duck circovirus in China, 2022. PLoS ONE. (2025) 20:e0323282. doi: 10.1371/journal.pone.0323282 40344561 PMC12064196

[3] Wang D Xie X Zhang D Ma G Wang X Zhang D. Detection of duck circovirus in China: a proposal on genotype classification. Vet Microbiol. (2011) 147:410–5. doi: 10.1016/j.vetmic.2010.07.014 20709471

[4] Xiang QW Zou JF Wang X Sun YN Gao JM Xie ZJ . Identification of two functional nuclear localization signals in the capsid protein of duck circovirus. Virology. (2013) 436:112–7. doi: 10.1016/j.virol.2012.10.035 23174505

[5] Xiang QW Wang X Xie ZJ Sun YN Zhu YL Wang SJ . ORF3 of duck circovirus: a novel protein with apoptotic activity. Vet Microbiol. (2012) 159:251–6. doi: 10.1016/j.vetmic.2012.03.045 22537707

[6] Huang J Zhang Y Cheng A Wang M Liu M Zhu D . Duck Circovirus genotype 2 ORF3 protein induces apoptosis through the mitochondrial pathway. Poult Sci. (2023) 102:102533. doi: 10.1016/j.psj.2023.102533 36848756 PMC9984893

[7] Zhang T Liu N Zhang L Jiang W Fan X Wang X . Research note: complete genome cloning and genetic evolution analysis of four Cherry Valley duck circovirus strains in China in 2022. Poult Sci. (2023) 102:102920. doi: 10.1016/j.psj.2023.102920 37473522 PMC10371810

[8] Hattermann K Schmitt C Soike D Mankertz A. Cloning and sequencing of Duck circovirus (DuCV). Arch Virol. (2003) 148:2471–80. doi: 10.1007/s00705-003-0181-y 14648300

[9] Liao JY Xiong WJ Tang H Xiao CT. Identification and characterization of a novel circovirus species in domestic laying ducks designated as duck circovirus 3 (DuCV3) from Hunan province, China. Vet Microbiol. (2022) 275:109598. doi: 10.1016/j.vetmic.2022.109598 36332301

[10] Chen CL Wang PX Lee MS Shien JH Shien HK Ou SJ . Development of a polymerase chain reaction procedure for detection and differentiation of duck and goose circovirus. Avian Dis. (2006) 50:92–5. doi: 10.1637/7435-090705R1.1 16617989

[11] Lei X Wang A Zhu S Wu S. From obscurity to urgency: a comprehensive analysis of the rising threat of duck circovirus. Vet Res. (2024) 55:12. doi: 10.1186/s13567-024-01265-2 38279181 PMC10811865

[12] Zhang Z Jia R Wang M Lu Y Zhu D Chen S . Complete genome sequence of the Novel Duck Circovirus Strain GH01 from Southwestern China. Genome Announc. (2013) 1:e00166–12. doi: 10.1128/genomeA.00166-12 23405313 PMC3569302

[13] Xie L Xie Z Zhao G Liu J Pang Y Deng X . Complete genome sequence analysis of a duck circovirus from Guangxi pockmark ducks. J Virol. (2012) 86:13136. doi: 10.1128/JVI.02494-12 23118461 PMC3497624

[14] Wang X Li L Shang H Zhou F Wang C Zhang S . Effects of duck circovirus on immune function and secondary infection of Avian Pathogenic Escherichia coli. Poult Sci. (2022) 101:101799. doi: 10.1016/j.psj.2022.101799 35366422 PMC8971308

[15] Xu S Man Y Xu X Ji J Mu X Yao L . Genetic heterogeneity of duck circovirus first detected in geese from China. Poult Sci. (2024) 103:104284. doi: 10.1016/j.psj.2024.104284 39293260 PMC11426040

[16] Li Z Wang X Zhang R Chen J Xia L Lin S . Evidence of possible vertical transmission of duck circovirus. Vet Microbiol. (2014) 174:229–32. doi: 10.1016/j.vetmic.2014.09.001 25263494

[17] Hong YT Kang M Jang HK. Pathogenesis of duck circovirus genotype 1 in experimentally infected Pekin ducks. Poult Sci. (2018) 97:3050–7. doi: 10.3382/ps/pey177 29788411

[18] Stenzel T Dziewulska D Muhire BM Hartnady P Kraberger S Martin DP . Recombinant goose circoviruses circulating in domesticated and wild geese in Poland. Viruses. (2018) 10:107. doi: 10.3390/v10030107 29498637 PMC5869500

[19] Kumar S Stecher G Li M Knyaz C Tamura K Mega X. Molecular evolutionary genetics analysis across computing platforms. Mol Biol Evol. (2018) 35:1547–9. doi: 10.1093/molbev/msy096 29722887 PMC5967553

[20] Ji J Chen Q Sui C Yu Z Xu X Yao L . Novel genotype definition and genome characteristics of duck circovirus in central and Eastern China. Transbound Emerg Dis. (2020) 67:2993–3004. doi: 10.1111/tbed.13676 32531142

[21] Li P Li J Zhang R Chen J Wang W Lan J . Duck “beak atrophy and dwarfism syndrome” disease complex: interplay of novel goose parvovirus-related virus and duck circovirus. Transbound Emerg Dis. (2018) 65:345–51. doi: 10.1111/tbed.12812 29341432

[22] Matczuk AK Krawiec M Wieliczko A A. new duck circovirus sequence, detected in velvet scoter (*Melanitta fusca*) supports great diversity among this species of virus. Virol J. (2015) 12:121. doi: 10.1186/s12985-015-0352-y 26253134 PMC4528844

[23] Tan C Wang Z Lei X Lu J Yan Z Qin J . Epidemiology, molecular characterization, and recombination analysis of chicken anemia virus in Guangdong province, China. Arch Virol. (2020) 165:1409–17. doi: 10.1007/s00705-020-04604-8 32318833

