# Supplementary material for: Genetic variability and intra-genotype recombination of DuCV from ducks and geese in central and north China
Source: Front Vet Sci. 2026 Apr 13;13:1775879. doi: 10.3389/fvets.2026.1775879 (PMC13113269; doi:10.3389/fvets.2026.1775879)
Supplement: Supplementary file 1 [file Table_1.docx]

Supplementary Material

# Genetic variability and intra-genotype recombination of DuCV from ducks and geese in Central and North China

# Haoyang Wang^1^, Yuzhu Dong^1^, Xiaomin Hu^1^, Jinglan Wang^2^, Xin Xu^1^, Dandan Li^1^, Jun Ji^1,^*, Lunguang Yao^1^, Yingzuo Bi^3^, Qingmei Xie^3^

^1^Henan Provincial Engineering Laboratory of Insects Bio-reactor, Henan Provincial Engineering, and Technology Center of Health Products for Livestock and Poultry, Henan Provincial Engineering and Technology Center of Animal Disease Diagnosis and Integrated Control, Nanyang Normal University, Nanyang, 473061, PR China;

^2^Langfang Academy of Agricultural and Forestry Sciences, Langfang, 065000, PR China;

^3^College of Animal Science, South China Agricultural University, Guangzhou 510642, PR China.

**Correspondence:** Corresponding Author: [jijun020@126.com](mailto:jijun020@126.com)

**Supplementary Table 1.** The cycling condition for PCR assay

| Temperature | Time | Cycle | Function |
| --- | --- | --- | --- |
| 94℃ | 3 min | 1 | Pre-denaturation |
| 94℃ | 30 s | 30 | Denaturation |
| 51℃ (DuCV1/2) | 30 s | 30 | Annealing |
| 55 ℃ (DuCV3) |  |  |  |
| 57℃ (DuCV) |  |  |  |
| 72℃ | 30 s (DuCV1/2) | 30 | Extension |
|  | 2 min (DuCV3) |  |  |
|  | 2 min (DuCV) |  |  |
| 72℃ | 10 min | 1 | Final extension |

**Supplementary Table 2.** Information on identified goose- and duck-derived DuCV strains

| **Name** | **Collection Source** | **Host** | **Accession no.** | **Year** | **Province** |
| --- | --- | --- | --- | --- | --- |
| D250105 | Liver and spleen | Duck | PX308121 | 2025 | Hubei |
| D250507 | Liver and spleen | Duck | PX308123 | 2025 | Hebei |
| D250425 | Liver and spleen | Duck | PX308124 | 2025 | Hebei |
| D250106 | Liver and spleen | Duck | PX308125 | 2025 | Hebei |
| D250207 | Liver and spleen | Duck | PX308126 | 2025 | Hubei |
| D230823 | Liver and spleen | Duck | PX308127 | 2023 | Henan |
| D240605 | Liver and spleen | Duck | PX308129 | 2024 | Henan |
| D240110 | Liver and spleen | Duck | PX308132 | 2024 | Henan |
| D240923 | Liver and spleen | Duck | PX308133 | 2024 | Henan |
| G250408 | Liver and spleen | Goose | PX308122 | 2025 | Henan |
| G231113 | Liver and spleen | Goose | PX308128 | 2023 | Hubei |
| G240828 | Liver and spleen | Goose | PX308130 | 2024 | Hubei |
| G241017 | Liver and spleen | Goose | PX308131 | 2024 | Hebei |

**Supplementary Table 3.** Information of reference strains employed in this study.

| Strains | Accession Nos. | Host | Type |
| --- | --- | --- | --- |
| FujianZQ300 | GQ423740.1 | Duck | DuCV-1a |
| FJzq290 | GU168779.1 | Duck | DuCV-1a |
| ZC03 | EU344807.1 | Duck | DuCV-1a |
| YS07 | EU344806.1 | Duck | DuCV-1a |
| AH03 | MN928810.1 | Duck | DuCV-1a |
| DU101 | HM162350.1 | Duck | DuCV-1a |
| FJMH207 | GQ423742.1 | Duck | DuCV-1a |
| LJ33 | EU344803.1 | Duck | DuCV-1a |
| LJ07 | EU499311.1 | Duck | DuCV-1a |
| MH06 | EU344804.1 | Duck | DuCV-1a |
| MH25 | EF451157.1 | Duck | DuCV-1a |
| WF0801 | GU131340.1 | Duck | DuCV-1a |
| GX190511 | MK814582.1 | Duck | DuCV-1a |
| GX190512 | MK814583.1 | Duck | DuCV-1a |
| wd2013046 | KU844856.1 | Duck | DuCV-1a |
| DU091 | HM162345.1 | Duck | DuCV-1a |
| Zhejiang | GQ334371.1 | Duck | DuCV-1a |
| FJZZ302 | GQ423747.1 | Duck | DuCV-1a |
| ZJ03 | MN928797.1 | Duck | DuCV-1b |
| HN04 | MN928798.1 | Duck | DuCV-1b |
| TA0914 | OQ657186.1 | Duck | DuCV-1b |
| DZ1116 | OQ657184.1 | Duck | DuCV-1b |
| Vietnam/VNUA-HN47/2021 | OM176553.1 | Duck | DuCV-1b |
| Vietnam/VNUA-TB61/2021 | OM176554.1 | Duck | DuCV-1b |
| D11-JW-001 | JQ740360.1 | Duck | DuCV-1b |
| D11-JW-004 | KC851804.1 | Duck | DuCV-1b |
| D11-JW-007 | KC851805.1 | Duck | DuCV-1b |
| D11-JW-009 | KC851806.1 | Duck | DuCV-1b |
| D11-JW-025 | KC851812.1 | Duck | DuCV-1b |
| D11-JW-037 | KC851813.1 | Duck | DuCV-1b |
| D12-KD-002 | KC851818.1 | Duck | DuCV-1b |
| D12-KD-027 | KC851820.1 | Duck | DuCV-1b |
| WF0706 | OQ657183.1 | Duck | DuCV-1b |
| JS0807 | OQ657185.1 | Duck | DuCV-1b |
| GD/ZQ/141 | ON227545.1 | Duck | DuCV-1b |
| HN02 | MN928795.1 | Duck | DuCV-1b |
| Vietnam/VNUA-TN85/2021 | OM176555.1 | Duck | DuCV-1b |
| JSPX03E | MF627688.1 | Duck | DuCV-1b |
| GX190509 | MK814580.1 | Duck | DuCV-1b |
| GX190510 | MK814581.1 | Duck | DuCV-1b |
| ZJ01 | MN928792.1 | Duck | DuCV-1b |
| AH04 | MN928811.1 | Duck | DuCV-1b |
| AH01 | MN928808.1 | Duck | DuCV-1b |
| AH02 | MN928809.1 | Duck | DuCV-1b |
| TD/41/09 | HQ180266.1 | Duck | DuCV-1b |
| G210917 | PP950795.1 | Goose | DuCV-1b |
| ZJ02 | MN928793.1 | Duck | DuCV-1b |
| HN05 | MN928799.1 | Duck | DuCV-1b |
| LQ-1-2022 | ON756212.1 | Duck | DuCV-1b |
| SDTZ2161 | OP850042.1 | Duck | DuCV-1b |
| SDLY0201 | MF627690.1 | Duck | DuCV-1b |
| SDDY0520 | MF627689.1 | Duck | DuCV-1b |
| DB7-17 | KP229364.1 | Duck | DuCV-1c |
| AQ0901 | GU014543.1 | Duck | DuCV-1c |
| CD13056 | KP229375.1 | Duck | DuCV-1c |
| FJ06 | MN928807.1 | Duck | DuCV-1d |
| FJ03 | MN928804.1 | Duck | DuCV-1d |
| FJ02 | MN928803.1 | Duck | DuCV-1d |
| FJ01 | MN928802.1 | Duck | DuCV-1d |
| FJ05 | MN928806.1 | Duck | DuCV-1d |
| AHAU25 | MT646347.1 | Duck | DuCV-1d |
| YN190412 | MK814588.1 | Duck | DuCV-1d |
| YN190415 | MK814589.1 | Duck | DuCV-1d |
| YN190411 | MK814587.1 | Duck | DuCV-1d |
| YN26-2013 | KR491946.1 | Duck | DuCV-1d |
| YN190410 | MK814586.1 | Duck | DuCV-1d |
| HZ09 | EU344802.1 | Duck | DuCV-2a |
| MH11 | EU344805.1 | Duck | DuCV-2a |
| MH02/07 | EU499309.1 | Duck | DuCV-2a |
| Vietnam/VNUA-BG135/2021 | OM176557.1 | Duck | DuCV-2b |
| CP12021 | KP229377.1 | Duck | DuCV-2b |
| wd2014012 | KU844857.1 | Duck | DuCV-2b |
| TC3/2002 | DQ166837.1 | Duck | DuCV-2b |
| TC4/2002 | DQ166838.1 | Duck | DuCV-2b |
| TC2/2002 | DQ166836.1 | Duck | DuCV-2b |
| TC1/2002 | AY394721.1 | Duck | DuCV-2b |
| NC-006561.1 | NC-006561.1 | Duck | DuCV-2b |
| AHAU37 | MT646349.1 | Duck | DuCV-2c |
| G221116 | PP950796.1 | Goose | DuCV-2c |
| GD/JM/A | ON227555.1 | Duck | DuCV-2c |
| GD/QY/B | ON227556.1 | Duck | DuCV-2c |
| GD/ZQ/98 | ON227536.1 | Duck | DuCV-2c |
| GD/FS/C | ON227557.1 | Duck | DuCV-2c |
| WS-GD01 | FJ554673.1 | Duck | DuCV-2c |
| FJ0701 | GQ868757.1 | Duck | DuCV-2c |
| WF0804 | GU131343.1 | Duck | DuCV-2c |
| WF0802 | GU131341.1 | Duck | DuCV-2c |
| LY0701 | EU022374.1 | Duck | DuCV-2c |
| FJ0601 | EF370476.1 | Duck | DuCV-2c |
| GD190402 | MK814576.1 | Duck | DuCV-2c |
| GD180503 | MK814573.1 | Duck | DuCV-2c |
| GD180502 | MK814572.1 | Duck | DuCV-2c |
| GD180504 | MK814574.1 | Duck | DuCV-2c |
| GD180501 | MK814571.1 | Duck | DuCV-2c |
| GD190401 | MK814575.1 | Duck | DuCV-2c |
| GD190403 | MK814577.1 | Duck | DuCV-2c |
| wd2015028 | KU844858.1 | Duck | DuCV-2c |
| Vietnam/VNUA-HD89/2021 | OM176556.1 | Duck | DuCV-2c |
| YN180505 | MK814584.1 | Duck | DuCV-2c |
| YN180506 | MK814585.1 | Duck | DuCV-2c |
| AHAU28 | MT646348.1 | Duck | DuCV-2c |
| FJPT09 | GQ423741.1 | Duck | DuCV-2c |
| GH01 | JX499186.1 | Duck | DuCV-2c |
| DuCV3/Duck/CHN/2022/HNU-HYH | OP432310.1 | Duck | DuCV-3 |
